# Resolvin D5 Protects Female Hairless Mouse Skin from Pathological Alterations Caused by UVB Irradiation

**DOI:** 10.3390/antiox13081008

**Published:** 2024-08-19

**Authors:** Priscila Saito, Ingrid C. Pinto, Camilla C. A. Rodrigues, Ricardo L. N. de Matos, David L. Vale, Cristina P. B. Melo, Victor Fattori, Telma Saraiva-Santos, Soraia Mendes-Pierotti, Mariana M. Bertozzi, Ana P. F. R. L. Bracarense, Josiane A. Vignoli, Marcela M. Baracat, Sandra R. Georgetti, Waldiceu A. Verri, Rubia Casagrande

**Affiliations:** 1 Departamento de Ciências Farmacêuticas, Universidade Estadual de Londrina, Avenida Robert Koch, 60, Hospital Universitário, Londrina 86039-440, Paraná, Brazil; prsaito@gmail.com (P.S.); carol.ingrid2@gmail.com (I.C.P.); camilla_arriero@hotmail.com (C.C.A.R.); ricardolnm@uel.br (R.L.N.d.M.); dlvale31@msn.com (D.L.V.); cristinadepaulamelo@gmail.com (C.P.B.M.); soraia.pierotti@uel.br (S.M.-P.); baracat@uel.br (M.M.B.); srgeorgetti@uel.br (S.R.G.); 2 Departamento de Imunologia, Parasitologia e Patologia Geral, Centro de Ciências Biológicas, Universidade Estadual de Londrina, Rodovia Celso Garcia Cid, Km 80, PR445, Cx. Postal 10.011, Londrina 86057-970, Paraná, Brazil; victor.fattori@childrens.harvard.edu (V.F.); telma@wustl.edu (T.S.-S.); mariana.mbertozzi@uel.br (M.M.B.); waverri@uel.br (W.A.V.); 3 Laboratório de Patologia Animal, Universidade Estadual de Londrina, Campus Universitário, Rodovia Celso Garcia Cid, Km 380, Londrina 86057-970, Paraná, Brazil; anapaula@uel.br; 4 Departamento de Bioquímica e Biotecnologia, Centro de Ciências Exatas, Universidade Estadual de Londrina, Rodovia Celso Garcia Cid, Km 380, Londrina 86057-970, Paraná, Brazil; javignoli@uel.br

**Keywords:** collagen, cytokine, inflammation, lipid, oxidative stress, reactive oxygen species, resolvin D5, skin, specialized pro-resolution lipid mediator, skin, UVB

## Abstract

Resolvin D5 (RvD5) is a lipid mediator that has been reported to present anti-inflammatory and pro-resolution properties. Evidence also supports its capability to enhance reactive oxygen species (ROS) production during bacterial infections, which would be detrimental in diseases driven by ROS. The biological activity of RvD5 and mechanisms against UVB irradiation skin pathology have not been investigated so far. Female hairless mice were treated intraperitoneally with RvD5 before UVB stimulus. RvD5 reduced skin edema in a dose-dependent manner as well as oxidative stress by increasing antioxidants (endogenous tissue antioxidant scavenging of cationic radical, iron reduction, catalase activity and reduced glutathione levels) and decreasing pro-oxidants (superoxide anion and lipid peroxidation). RvD5 antioxidant activity was accompanied by enhancement of Nrf2, HO-1 and NQO1 mRNA expression. RvD5 reduced the production of IL-1β, TNF-α, TGF-β, and IL-10. RvD5 also reduced the inflammatory cell counts, including mast cells and neutrophils/macrophages. The reduction of oxidative stress and inflammation resulted in diminished matrix metalloproteinase 9 activity, collagen degradation, epidermal thickening and sunburn cell development. Therefore, this study demonstrates, to our knowledge, the first body of evidence that RvD5 can be used to treat UVB skin pathology and unveils, at least in part, its mechanisms of action.

## 1. Introduction

The skin is the largest organ in the body, comprising 16% of our body mass; however, its integrity is threatened by sunlight exposure [1]. Ultraviolet (UV) irradiation is a leading cause of skin damage [2,3,4] and has been associated with photoaging, immunomodulation and, importantly, skin cancer [5,6]. Oxidative stress is a major mechanism of UV irradiation activity. On the other hand, our body has endogenous antioxidant defenses, for instance, reduced glutathione (GSH) and catalase, which can be depleted depending on the intensity of UVB irradiation challenge. The depletion of endogenous antioxidants occurs by excessive production of reactive oxygen species (ROS) such as superoxide anion causing lipid peroxidation [7,8]. Superoxide anion is an endogenous ROS that triggers the development of inflammatory edema and leukocyte recruitment [9]. Furthermore, ROS and cytokines induce the apoptosis of keratinocytes, which are known as sunburn cells, and their presence is one of the characteristic histopathological cellular modifications caused by ultraviolet B irradiation (UVB). Erythema, edema and histological changes such as thickening of the epidermis, and infiltration of inflammatory cells such as neutrophils and monocytes/macrophages are also characteristic histopathological modifications triggered by UVB [10].

Superoxide anion induces the production of cytokines such as TNFα [11]. This is a pro-inflammatory cytokine that orchestrates neutrophil recruitment in inflammation in varied disease conditions including UV skin inflammation [12]. TNFα is one of the endogenous inflammatory molecules responsible for sunburn cell induction caused by UVB [13]. Furthermore, TNFα stimulates the production of ROS, thus, amplifying inflammation and oxidative stress [14,15]. Other inflammatory cytokines such as IL-1β participate in these processes, which are counter-regulated by IL-10 and TGFβ [16]. These are anti-inflammatory cytokines that are co-released with pro-inflammatory cytokines during the inflammatory process, then, the production of IL-10 and TGFβ is not necessary if inflammation does not occur [17]. The balance between pro- and anti-inflammatory mediators will dictate the intensity of inflammation, the extent of cellular activation, cellular recruitment and tissue damage [18].

In essence, inflammation serves as a protective response against infections and to clear dead cells and cellular debris facilitating the process of tissue repair. However, excessive inflammation or a response targeting autoantigens cause tissue damage, which may lead to inflammation becoming the disease itself [19,20]. Our organism, nonetheless, evolved to limit inflammation in an active manner. Among the endogenous molecules regulating inflammation resolution, we highlight the specialized pro-resolving lipid mediators (SPMs) [21] that encompass four main classes of molecules: the lipoxins, resolvins, protectins and maresins. Our group was the first to describe that an SPM can reduce pathological alterations in the skin caused by UVB irradiation by treating hairless mice with lipoxin A4 [22]. We also have shown that resolvin D1, aspirin-triggered RvD1 and maresin 1 reduce skin pathology caused by UVB irradiation [23,24,25] as well as other lipids such as 15d-prostaglandin J2 [26]. Thus, we propose the concept that such SPMs can be used as pharmacological treatments against UVB irradiation pathological alterations in the skin.

Resolvin D5 (RvD5) is an SPM derived from docosahexaenoic acid (DHA) metabolization by an enzyme called 5-lipooxygenase [27]. Evidence demonstrates RvD5 is an agonist of the human GPR32 receptor, which is also named RvD1 receptor; however, there is no identified murine homolog for this receptor [28,29]. Interestingly, an N-3 docosapentaenoic-acid-derived resolvin D5 (RvD5_n-3 DPA_) acts through GPR101, which is also a receptor for RvD5 [30]. The deletion of GPR101 enhances inflammation, demonstrating the endogenous limiting role of agonists of this receptor in regulating inflammation [31]. The study on the biological activities of RvD5 is still an evolving field with limited literature. What else is known about the activities and mechanisms of RvD5? This SPM reduces, by diminishing IL-1β production in the pre-frontal cortex and hippocampus, the anxious and depressive-like behaviors in a rat model of diabetes mellitus [32]. RvD5 can shift macrophage phenotype from an inflammatory towards non-inflammatory macrophage phenotype [33]. It is indeed interesting that RvD5 can enhance *Escherichia coli* phagocytosis and killing by increasing phagocyte ROS production, and at the same time reduce the release of pro-inflammatory cytokines such as TNFα by a phagocyte that actively kills bacteria [29]. This cell type is called a resolution macrophage since it is still protective against infections but does not enhance inflammation [28]. In another bacterial infection model, infant *Citrobacter rodentium* infection, RvD5, again, reduced bacterial load, inflammation, and infection lethality [34]. Thus, RvD5 can reduce inflammation without causing immunosuppression, making it ideal to treat bacterial infections [28]. Intriguingly, trypomastigotes forms of *Trypanossoma cruzi* can produce RvD5 although its endogenous role has not been elucidated in Chagas disease [35]. In a scenario without infection and with Th17 cells akin to that observed in rheumatoid arthritis, RvD5 has a role in reshaping such adaptive immune response. In zymosan arthritis in SKG mice, the balance between Th17 cells and regulatory T cells was changed towards regulatory T cells by RvD5 treatment, which also reduced osteoclastogenesis [36]. Therefore, the inhibition of the production of pro-inflammatory mediators by RvD5 seems also to be a consistent mechanism of action. For instance, RvD5 reduces the production of inflammatory cytokines by targeting ERK and NFkB in THP-1 cells stimulated by LPS [27]. In addition, a mixture of 17S-monohydroxy DHA, resolvin D5, and protectin DX obtained by the metabolization of DHA by soybean lipoxygenase inhibits atopic dermatitis skin inflammation by inhibiting cytokine production in vivo. In vitro, it was observed that this lipid mediator mixture targets NFκB in HaCaT cells stimulated with TNF-α/IFN-γ to reduce cytokine production [37]. Although this study applied a mixture of lipid mediators, it highlights that there are biotechnological efforts towards developing SPM-based biotherapeutic products including RvD5 as a relevant active molecule.

It is also interesting to point out that in terms of the analgesia provided by RvD5, evidence supports that it is active in male mice, but not in female mice in models of chemotherapy-induced neuropathy and formalin nociception [38]. Male rats also demonstrated greater sensitivity to the analgesic activity of RvD5 compared to female rats in a model of chronic constriction injury of the infraorbital nerve [39]. Thus, overall, there is no question on whether RvD5 is active in male rodents, but rather if it is active in female rodents [40]. In this sense, the current literature indicates that it is important to determine whether RvD5 is active in females or not, and in which scenarios it is active in females.

Therefore, the current evidence supports that RvD5 can reduce the production of inflammatory mediators [27]. During bacterial infections, RvD5 increases host anti-microbial response, which is good for infection resolution. However, increasing ROS production that is an antimicrobial defense mechanism [29] would be detrimental in UVB irradiation pathology [41,42]. We reason, therefore, that investigating the pharmacological activity of RvD5 in a model of sterile inflammation such as UVB irradiation skin inflammation, which is an ROS-dependent pathology, is necessary to better understand its biological properties and eventual side effects. This is especially relevant in UVB irradiation inflammation since it is initiated by ROS production [8]. Another starting point would be whether RvD5 is active in female hairless mice due to prior studies demonstrating that this SPM confers little or no analgesic effects in female rodents in contrast to their male counterparts [38,39]. To our knowledge, there is no study investigating the activity and mechanisms of RvD5 in UVB irradiation skin pathology to date.

## 2. Materials and Methods

### 2.1. Chemicals

Chemicals were obtained from the following sources: Resolvin D5 from Cayman Chemical (Ann Arbor, MI, USA); brilliant blue R, reduced glutathione (GSH), hexadecyltrimethylammonium bromide (HTAB), o-dianisidine dihydrochloride, 5,5′-dithiobis (2-nitrobenzoic acid) (DTNB), nitroblue tetrazolium (NBT), and bisacrylamide from Sigma-Aldrich (St. Louis, MO, USA); tert-butyl hydroperoxide from Acros (Pittsburgh, PA, USA); tris from Amresco (Solon, OH, USA); ELISA kits for the determination of cytokine from eBioscience (San Diego, CA, USA); and acrylamide, sodium dodecyl sulfate (SDS), platinum SYBRGreen, and superscript III kits from Invitrogen (Waltham, MA, USA). All other reagents used were of pharmaceutical grade.

### 2.2. Animals

The experiments were performed in female hairless mice (HRS/J) weighing 20–30 g obtained from the University Hospital of Londrina State University (UEL), Paraná, Brazil or female LysM-eGFP^+^ mice of C57BL/6 background (weighing 20–25 g) obtained from Ribeirao Preto Medical School, University of Sao Paulo, SP, Brazil.

The mice were maintained with free access to water and food throughout the experiment with a light/dark cycle of 12/12 h and temperature-controlled (23 ± 2 °C). The Animal Ethics Committee of the Londrina State University approved all procedures used in this study (CEUA process number 11146.2016.97). All methods were performed following the relevant guidelines and regulations. All experiments were performed with the minimum number of animals and minimum suffering. In this sense, euthanasia was performed at the end of experiments by terminal anesthesia with isoflurane 5% (Abbott Park, IL, USA) followed decapitation. Animals were used only once. The mice were continuously monitored regarding welfare-related assessment. LysM-eGFP+ mice were used in the experiment for evaluating the participation of LysM^+^ leukocytes in the inflammatory process of skin after UVB exposure. LysM-eGFP^+^ strain produces a green fluorescent protein under lysozyme M promoter (LysM) control, which is an enzyme expressed in neutrophils and macrophages [24,43].

### 2.3. Experimental Protocol

Female hairless mice were randomly assigned to six groups with six animals per group. The groups were: non-irradiated control treated with vehicle (saline), irradiated control treated with vehicle (saline, which was indicated as 0), irradiated treated with RvD5 3 pg/mouse, irradiated treated with RvD5 10 pg/mouse, and irradiated treated with RvD5 30 pg/mouse. RvD5 dilutions were prepared with saline solution. The doses of RvD5 used in the treatments were selected based on dose-response curves tested in the present study. Considering the literature question on whether RvD5 is active or not in female rodents, we reason that testing it first in female mouse would address this issue, and upon detecting activity in female mouse, we should continue with the protocols in female mouse.

Mice were treated with RvD5 (via intraperitoneal administration) 1 h before and 7 h after the beginning of UVB irradiation exposure [23]. Animals in the control groups received treatment with vehicle (saline).

After the UVB irradiation exposure, samples of skin mice were dissected 2 h, 4 h, or 12 h after the exposure, depending on the assay. Each parameter was evaluated at a specific time point, which was previously determined as suitable to detect significant differences between non-irradiated control and irradiated control groups [44,45,46], therefore being valid for determining possible treatment effects with RvD5.

The results obtained in the assays evaluating skin edema, FRAP, ABTS, and GSH were utilized for selecting the optimal dose of RvD5. This dose was used in the following assays to analyze the effect of RvD5 on UV-induced oxidative stress (hydroperoxide formation and superoxide anion production) and determine Nrf2, NQO1, and HO-1 mRNA expression by RT-qPCR. The optimal dose of RvD5 was also used to evaluate inflammatory (IL-1β and TNF-α) and anti-inflammatory (TGF-β and IL-10) cytokine levels, histopathological alterations (epidermal thickness, sunburn cell counts, collagen degradation, and mast cell counts), and MMP-9 activity. The optimal dose of RvD5 was also used for experiments involving LysM-eGFP^+^ mice. They were randomly assigned to five mice per group and treated with the protocol described previously.

### 2.4. Irradiation Protocol

UVB lamp (Philips TL/12 RS 40 W, Medical-Holand) was used in the experiments to induce oxidative stress and acute inflammatory process in hairless mice or LysM-eGFP^+^ mice. The lamp emits light between 270 and 400 nm with a peak emission at 313 nm. The radiation dose used to induce process inflammatory and oxidative stress was 4.14 J/cm^2^ [22].

The lamp was mounted 20 cm overhead the table where the mice were allocated, and all animals were irradiated simultaneously, as previously described [47]. It is important to mention that each mouse was used only once, and euthanasia procedure was performed before skin tissue collection. At 12h after the irradiation, the hairless mice or Lysm-eGFP^+^ mice were terminally anesthetized with 5% isoflurane (Abbott [Abbott Park, IL, USA]). The full thickness of the dorsal skins was removed for edema, MMP-9 activity, GSH assays, histology analysis, and immunofluorescence assay. For catalase and NBT assays, the hairless mice were anesthetized with 5% isoflurane, followed by decapitation 2h after the end of irradiation. To evaluate hydroperoxides, cytokines and mRNA expression, mice were anesthetized with 5% isoflurane, followed by decapitation 4h after the end of irradiation. After euthanasia, dorsal skin was removed. Each parameter was evaluated at a specific time, which was previously determined [22,47].

After collection, the skin samples were stored at −80 °C until analysis. For analysis of cutaneous edema, the samples were weighed immediately after collection, for histological examination, the samples were fixed in buffered formaldehyde, and immunofluorescence assay, the samples were fixed in 4% paraformaldehyde (PFA) for 24 h.

### 2.5. Skin Edema

Dorsal skin samples were collected carefully with the aid of a 5 mm diameter mold. The results were expressed in mg of skin tissue obtained from the weight of each sample. The skin weight from the control group (irradiated and non-irradiated group) and treated group were compared [22,23].

### 2.6. Total Antioxidant Capacity: FRAP and ABTS Assays

For FRAP and ABTS assays, skin samples were dissected, homogenized into buffer containing 1.15% KCl, and centrifuged (1000× *g* in 4 °C for 10 min). Total antioxidant capacity was determined as described previously [46,48]. Reading was performed at 595 and 794 nm in a spectrophotometer reader (ENSPIRE, PERKIN ELMER). The results were expressed as nmol Trolox equivalent per mg of skin tissue. All results were compared to a standard curve of Trolox (concentration ranging between 0.01–20 nmol) [46,48].

### 2.7. Quantification of Endogenous Antioxidant: Reduced Glutathione (GSH)

GSH method is based on the quantification of the colored compound (5-mercapto-2-nitrobenzoic acid) formed by breaking the 5,5′-dithiobis (2-nitrobenzoic acid) (DTNB) bond by the glutathione sulfhydryl group [49].

GSH levels were determined as described previously [23]. The absorbance was performed at 405 nm. The results are presented as μM of GSH per mg of skin, compared to a standard curve (GSH concentration ranging 5–150 μM) [49].

### 2.8. Catalase Assay

Catalase activity was evaluated by measuring the decay of H_2_O_2_ concentration with resulting oxygen generation. Samples were homogenized in 500 μL of 0.02 M EDTA using a Tissue-Tearor (Biospec), and centrifuged twice (2700× *g*, 10 min, 4 °C). Ten μL of supernatants was mixed with 160 μL 1M Tris-HCl buffer with 5 mM EDTA (pH 8.0), 20 μL of deionized water, and 20 μL of 200 mM of H_2_O_2_. Catalase activity was determined at 25 °C through the difference between the initial reading and the reading conducted 30 s after the addition of H_2_O_2_ at 240 nm. Catalase values were expressed as units of catalase/mg of skin/minute [24].

### 2.9. Superoxide Anion Production

Exposure to UVB-induced formation of O_2_^•−^ by keratinocyte skin [50]. Production in the skin was measured using the nitroblue tetrazolium (NBT) assay as described previously [23]. Reduction of NBT to formazan was determined in spectrophotometer reader (ENSPIRE, PERKIN ELMER) at 620 nm, and the results are presented as NBT reduction (OD/10 mg of skin).

### 2.10. Lipid Peroxidation Assay (LPO)

LPO was evaluated by a chemiluminescence (QL) method previously described [23]. The method is based on the measure of the QL initiated by the tert-butyl hydroperoxide [51]. Dorsal skin samples were homogenized in 800 μL of Phosphate Buffered Saline (PBS) containing NaCl 137 mM, KCl 2.7 mM, Na_2_HPO_4_ 10 mM, KH_2_PO_4_ 1.8 mM pH 7.4 and centrifuged at 2000× *g*, for 2 min. The supernatant (70 μL) was mixed with 420 μL of reaction buffer (20 mM KH_2_PO_4_ with 0.9% NaCl pH 7.4), followed by addition of 10 μL of tert-butyl and 10 μL luminol. Each sample was analyzed for 3600 s resulting in the same quantity of points of chemiluminescence. Analysis of the reaction buffer without the sample was performed to check if there was interference in the chemiluminescence reading using the Glomax 20/20 (Madison, WI, USA). Results were expressed as relative light unit per mg of skin.

### 2.11. Cytokine Measurement by ELISA

The quantification of cytokines IL-1β, TNF-α, IL-10, and TGF-β in the skin was performed using commercial enzyme-linked immunosorbent assay (ELISA) kits according to manufacturer’s instructions (eBioscience). For that, skin samples were dissected and homogenized into sterile saline (500 µL). Reading was performed at 450 nm in a spectrophotometer reader (ENSPIRE, PERKIN ELMER), and the results are expressed as picograms (pg) of each cytokine/mg of skin tissue compared to the respective cytokine standard curve [52].

### 2.12. Real-Time and Quantitative Polymerase Chain Reaction (RT-qPCR)

The quantitative polymerase chain reaction method was described previously [23]. Briefly, skin samples were collected into Trizol reagent (Invitrogen), and total RNA was extracted as recommended by the manufacturer. RNA purity was determined spectrophotometrically in 260/280 nm (between 1.8 and 2.0 for all preparations). Reverse transcription of total RNA to cDNA and qPCR were performed using GoTaq^®^ 2-Step RT-qPCR System (Promega, Madison, WI, USA) on a StepOnePlus™ Real-Time PCR System (Applied Biosystems^®^, Thermo Fisher Scientific, Waltham, MA, USA). The relative gene expression was determined using the comparative 2^−(∆∆Ct)^ method, and GAPDH mRNA expression was used as a reference for tissue integrity in all samples. It is noteworthy to mention that GAPDH mRNA is stably expressed in the skin samples of naïve and UVB irradiated mice. Primer sequences were described previously [23].

### 2.13. Skin Histologic Evaluation

Skin samples were collected in formol 10%, embedded in paraffin, sectioned (5 μm), and stained with hematoxylin and eosin, toluidine blue, and Masson’s trichrome stain.

For epidermal thickness determination and sunburn cell count, the sections were stained with H & E and examined using light microscopy at 400× [53] and a 1000× magnification, respectively [13]. For mast cell count, the sections were stained with toluidine blue and analyzed under light microscopy at 400× magnification. All analyses were done with the software Infinity Analyze (Lumenera^®^ Software INFINITY CAPTURE Mac v. 6.3.1). Masson’s trichrome staining was also used to analyze the intensity of the blue coloration in the dermal areas of the skin exposed to UVB using light microscopy (100× magnification) with the aid of the ImageJ program (NIH, version 1.52q) as described previously [29]. Histopathological scores are presented together with the representative images quantifying the alterations detected between the groups. Statistical analysis was performed by one-way ANOVA followed by Tukey’s test [* *p* < 0.05 compared to the non-radiated control group; ^#^ *p* < 0.05 compared to the radiated control group (vehicle)].

### 2.14. Fluorescence Assay

LysM-eGFP^+^ mice were shaved 48 h before irradiation and treated according to the protocol outlined previously on topic 2.3. The dorsal samples of LysM-eGFP^+^ mice were immersed in 4% PFA and remained in this solution for 24 h. After this period, samples were transferred to a 30% sucrose solution for 24 h and to 30% sucrose solution with O.C.T. (1:1), before being included in O.C.T. The skin samples were sectioned in slides (thickness of 10 μm) and processed for fluorescence as described previously. The image was captured using a confocal microscope (Leica TCS SP8, Leica, Wetzlar, Germany) with a 5× objective. Images were processed using Leica EL6000 software (Leica, Wetzlar, Germany). The intensity of fluorescence was quantified in randomly selected fields (one field per sample, n = 5) of different groups by a blind evaluator. Result is presented as the percentage of eGFP fluorescence intensity [22].

### 2.15. Matrix Metalloproteinase (MMP)-9 Activity Measurement

Sodium dodecyl sulfate polyacrylamide gel electrophoresis (SDS-PAGE) substrate-embedded zymography was performed as described previously [29]. After electrophoresis, the gels were washed with 2.5% Triton X-100 with 0.05 M Tris-HCl (pH 7.4) for 1h on a rotary shaker to remove SDS and allow proteins to renature. The gels were then incubated in 0.01 M CaCl_2_ overnight at 37 °C, and stained with Brilliant Blue R. After destaining in 20% acetic acid, the proteolytic activity was analyzed by comparing the intensities of the bands of each group using the Image J Program (NIH, Bethesda, MD, USA). We analyzed 6 gels in total, and each one presented the results of a pool of 2 mice per group per gel summing up 12 animals per group.

### 2.16. Statistical Analysis

Statistical analysis was performed using GraphPad Prism 7 software (GraphPad Software Inc., San Diego, CA, USA). Data were analyzed by one-way analysis of variance (ANOVA) followed by Tukey multiple comparisons test. Results were presented as mean ± standard error (SEM) of measurements made with 6 animals in each group (5 animals for immunofluorescence) per experiment. The results were representative of 2 separate experiments and were considered significantly different at *p* < 0.05.

## 3. Results

### 3.1. Effect of Resolvin D5 (RvD5) against Skin Edema Caused by UVB Irradiation

**Figure 1:**
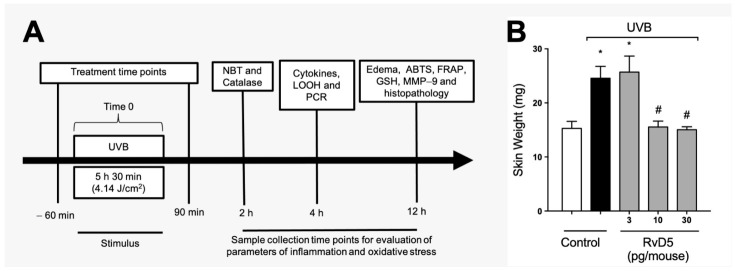
RvD5 reduces the development of skin edema induced by UVB irradiation. (**A**) Experimental protocol. (**B**) Results of edema are presented as tissue weight in milligrams of skin. Bars are representative of two separate experiments and represent means ± SEM of 6 mice per group per experiment. Statistical analysis was performed by one-way ANOVA followed by Tukey’s test [* *p* < 0.05 compared to the non-irradiated control group; ^#^ *p* < 0.05 compared to the irradiated control group (vehicle)].

Figure 1A depicts the experimental approach used, including treatment regimen and time points of tissue collection with the respective assay. These time points were standardized in our group’s previous studies and reflect when there is significant induction of each parameter by UVB irradiation [24,26,29,54]. Edema is a major inflammatory clinical sign; therefore, we assessed the effect of RvD5 (3, 10 and 30 pg/mouse, ip, 1 h pre-treatment and 7 h post-treatment) on the skin edema caused by UVB irradiation (Figure 1B). UVB irradiation caused significant skin edema and RvD5 reduced it. The doses of RvD5 of 10 and 30 pg/mouse were significantly active in reducing skin edema (Figure 1B). This first experiment demonstrates that RvD5 is active against a clinical sign of inflammation in diseases, the edema. Considering that ascertaining whether RvD5 is active in female rodents is a current literature aim in understanding the action of this SPM [38,39,40], this experiment was developed with a primary focus on female mice. RvD5 was active as shown in Figure 1B, therefore, the study was continued using female mice.

### 3.2. RvD5 Inhibits UVB Irradiation-Induced Depletion of Skin Antioxidants

**Figure 2:**
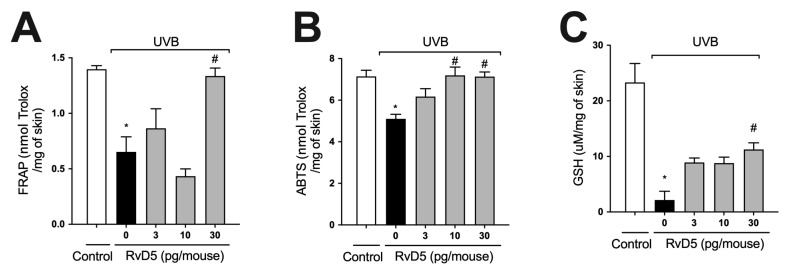
RvD5 inhibited UVB irradiation-induced decrease of skin antioxidant capacity. Protocol was followed as depicted in Figure 1A to investigate total antioxidant capacity FRAP (**A**), ABTS (**B**), and GSH levels (**C**). Results are presented as nmol of Trolox per milligrams of tissue for FRAP and ABTS assays and micromoles per milligrams of tissue for GSH assay. Bars are representative of two separate experiments and represent means ± SEM of 6 mice per group per experiment. Statistical analysis was performed by one-way ANOVA followed by Tukey’s test [* *p* < 0.05 compared to the non-irradiated control group; ^#^ *p* < 0.05 compared to the irradiated control group (vehicle)].

ROS are overproduced upon exposure to UVB irradiation and deplete antioxidant defenses leading to inflammation, oxidative stress and skin pathology [55,56]. Thus, the effect of treatment with RvD5 against UVB-induced oxidative stress was assessed. UVB irradiation led to decreased ferric reducing (Figure 2A) and ABTS scavenging (Figure 2B) tissue abilities. Also, it reduced GSH levels (Figure 2C) compared with the non-irradiated control group. All these antioxidant defenses that were reduced by UVB radiation were significantly reverted by the treatment with 30 pg of RvD5 per mouse. In addition, the treatment with 10 pg of RvD5 could prevent reduction in ABTS scavenging capacity. Based on the result obtained in edema (Figure 1B), FRAP, and GSH levels (Figure 2A,C), the dose of 30 pg/mouse was chosen for the next assays because it could inhibit all parameters.

### 3.3. RvD5 Interferes with ROS Metabolization, Production and Generation of Lipid Peroxidation End-Products in UVB Irradiation

**Figure 3:**
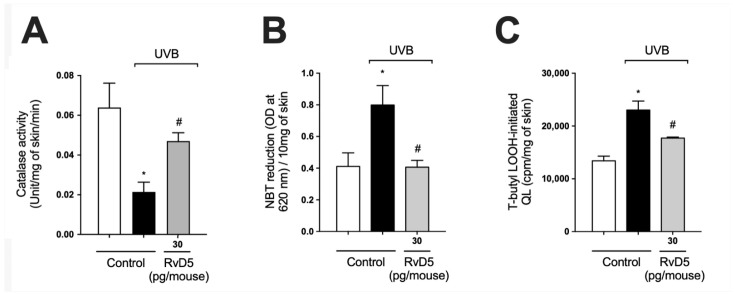
RvD5 inhibited UVB irradiation-induced decrease of skin catalase activity, and induction of superoxide anion production and lipid peroxidation. Protocol was followed as depicted in Figure 1A to investigate (**A**) catalase activity, (**B**) superoxide anion production, and (**C**) lipid peroxidation end-product LOOH. Bars are representative of two separate experiments and represent means ± SEM of 6 mice per group per experiment. Statistical analysis was performed by one-way ANOVA followed by Tukey’s test [* *p* < 0.05 compared to the non-irradiated control group; ^#^ *p* < 0.05 compared to the irradiated control group (vehicle)].

Catalase (CAT) degrades hydrogen peroxide into water and oxygen; thus, it is an important antioxidant enzyme of the skin. The decrease in levels of this enzyme upon UV-irradiation exposure represents potential damage to cellular macromolecules, as excess hydrogen peroxide can generate hydroxyl radical, a powerful oxidant capable of causing damage to all DNA bases [57]. UVB-irradiation decreased catalase activity in comparison with naïve control group. Our results showed that treatment with RvD5 restored catalase activity (Figure 3A). Skin exposure to UVB produces large amounts of reactive oxygen species (ROS) such as superoxide anion via mitochondrial complex I and III electron transport chain [24,58]. Exposure to UVB irradiation increased superoxide anion production comparable with non-irradiated control (Figure 3B). RvD5 treatment at 30 pg/mouse inhibited superoxide anion production. Oxidation of lipids can be measured by the formation of hydroperoxides (LOOH), which are the primary products in lipid peroxidation [59]. UVB irradiation increased the formation of LOOH and treatment with RvD5 30 pg/mouse reduced this production (Figure 3C). These data evidence that RvD5 reduces oxidative tissue damage (Figure 2 and Figure 3).

### 3.4. RvD5 Up-Regulates the mRNA Expression of Genes Involved in Skin Endogenous Antioxidant Response during UVB Irradiation Inflammation

**Figure 4:**
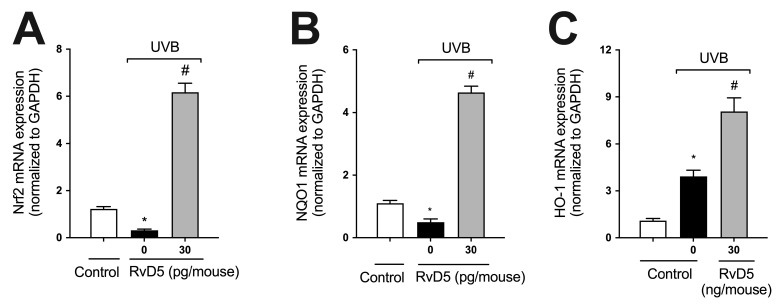
RvD5 enhances Nrf2, NQO1 and HO-1 mRNA expression in UVB irradiation. Protocol was followed as depicted in Figure 1A to investigate Nrf2 (**A**), NQO1 (**B**), and HO-1 (**C**) mRNA. Bars are representative of two separate experiments and represent means ± SEM of 6 mice per group per experiment. Statistical analysis was performed by one-way ANOVA followed by Tukey’s test. [* *p* < 0.05 compared to the non-irradiated control group; ^#^ *p* < 0.05 compared to the irradiated control group (vehicle)].

GSH (Figure 2C), NQO1, and HO-1 are downstream targets of Nrf2 [60]. We observed that UVB irradiation decreased the mRNA expression of both Nrf2 and NQO1 and increased that of HO-1, corroborating the findings of our previous study [29] (Figure 4). RvD5 treatment enhanced Nrf2, NQO1 and HO-1 mRNA expression (Figure 4) indicating this SPM up-regulates endogenous antioxidant/anti-inflammatory pathways [61].

### 3.5. RvD5 Inhibits UVB Irradiation-Induced Cytokine Production

**Figure 5:**
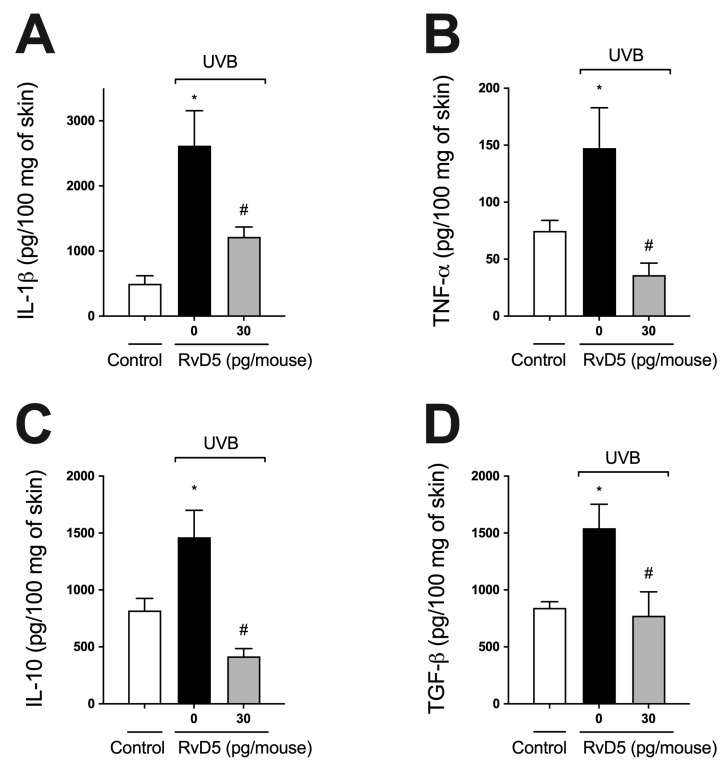
RvD5 inhibits UVB irradiation-induced cytokine production. Protocol was followed as depicted in Figure 1A to investigate IL-1β (**A**), TNFα (**B**), IL-10 (**C**) and TGFβ (**D**) production. Bars are representative of two separate experiments and represent means ± SEM of 6 mice per group per experiment. Statistical analysis was performed by one-way ANOVA followed by Tukey’s test [* *p* < 0.05 compared to the non-irradiated control group; ^#^ *p* < 0.05 compared to the irradiated control group (vehicle)].

Cytokines, such as IL-1β, TNF-α, TGF-β, and IL-10, are produced after overexposure to UVB radiation [22,24,29,54]. IL-1β and TNF-α are pro-inflammatory cytokines involved in various cellular and tissue alterations, including stimulation of ROS production and neutrophilic MMP-9 activity [16,62,63]. IL-10 and TGFβ down-modulate the production of pro-inflammatory cytokines and, consequently, the inflammatory response, besides orchestrating tissue repair [16,64]. RvD5 treatment reduced both pro- (Figure 5A,B) and anti-inflammatory cytokines (Figure 5C,D) induced by UVB irradiation (Figure 5).

### 3.6. RvD5 Reduces Mast Cell Counts in UVB Irradiation

**Figure 6:**
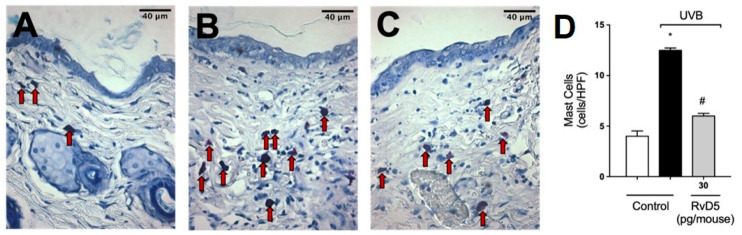
RvD5 reduced UVB irradiation-induced increase of mast cell count. Protocol was followed as depicted in Figure 1A to determine mast cells counts in toluidine blue stained slices. Representative images of the groups: non-irradiated control (**A**), irradiated treated with vehicle (**B**), and irradiated treated with 30 pg/mouse of RvD5 (**C**). Mast cells count of experimental groups is presented per field (**D**). Original magnification 400×. Bars are representative of two separate experiments and represent means ± SEM of 6 mice per group per experiment. Statistical analysis was performed by one-way ANOVA followed by Tukey’s test. [* *p* < 0.05 compared to the non-irradiated control group; ^#^ *p* < 0.05 compared to the irradiated control group (vehicle)]. Arrows indicate mast cells.

In the following sets of experiments, we addressed whether the inflammatory disease (e.g., edema), molecular, protein levels and enzymatic activity measurements presented in Figure 1, Figure 2, Figure 3, Figure 4 and Figure 5 would converge to down-regulation of skin pathology by RvD5 treatment. Exposure to UV radiation leads to increased recruitment of mast cells into the skin [65]. Mast cells secrete mediators that trigger inflammation and recruit other leukocytes such as neutrophils [66]. Treatment with RvD5 ameliorated UVB irradiation-induced mast cells counts in the skin (Figure 6).

### 3.7. RvD5 Treatment Inhibits Skin Recruitment of LysM^+^ Neutrophils/Macrophages in UVB Irradiation

**Figure 7:**
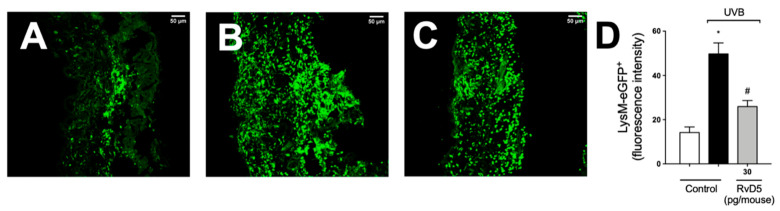
RvD5 reduces the recruitment of LysM-eGFP^+^ cells triggered by UVB irradiation. Protocol was followed as depicted in Figure 1A to investigate by fluorescence the recruitment of LysM-eGFP^+^ cells (neutrophils and macrophages) in the skin. Representative images of the groups: non-irradiated control treated with vehicle (salina) (**A**), irradiated treated with vehicle (saline) (**B**), and irradiated treated with 30 pg/mouse of RvD5 (**C**). Results are expressed in eGFP fluorescence intensity (**D**). Original magnification 20× (images **A**–**C**). Representative images from each group are presented with a 50 µm scale. Statistical analysis was performed by one-way ANOVA followed by Tukey’s test. [* *p* < 0.05 compared to the non-irradiated control group; ^#^ *p* < 0.05 compared to the irradiated control group (vehicle)].

We used LysM-eGFP^+^ mouse and fluorescence detection in a confocal microscope to determine the recruitment of neutrophils and macrophage to the skin in UVB irradiation (Figure 7). Exposure to UVB irradiation induced a robust infiltration of LysM^+^ neutrophils/macrophages compared to non-irradiated control, which was inhibited by RvD5 treatment.

### 3.8. RvD5 Inhibits MMP-9 Activity and Collagen Fibers Degradation in UVB Irradiation

**Figure 8:**
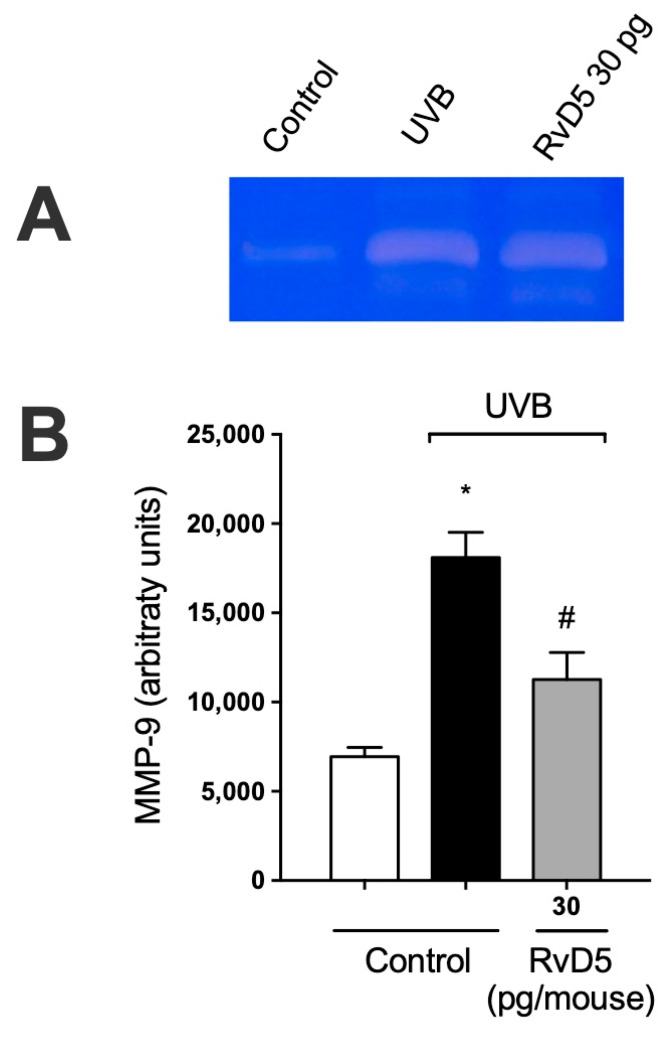
RvD5 inhibited UVB irradiation-induced MMP-9 activity in the skin. Protocol was followed as depicted in Figure 1A to investigate MMP-9 activity. (**A**) Representative image of gelatin zymography is presented. (**B**) Quantitation of skin MMP-9 activity. Results are presented as arbitrary units per sample for MMP-9 activity. Bars are representative of two separate experiments and represent means ± SEM of 6 mice per group per experiment. Statistical analysis was performed by one-way ANOVA followed by Tukey’s test. [* *p* < 0.05 compared to the non-irradiated control group; ^#^ *p* < 0.05 compared to the irradiated control group (vehicle)].

MMP-9 is an enzyme secreted predominantly by neutrophils, mast cells, and macrophages that has proteolytic activity against the main component of the basement membrane, type IV collagen [67,68,69,70]. MMP-9-induced damage to the collagenous matrix of the skin is one of the hallmarks of photoaging and non-melanoma skin cancer [71,72]. UVB irradiation increases MMP-9 activity and treatment with RvD5 reduces the activity of MMP-9. These data unveil that RvD5 reduces dermal connective tissue damage (Figure 8).

**Figure 9:**
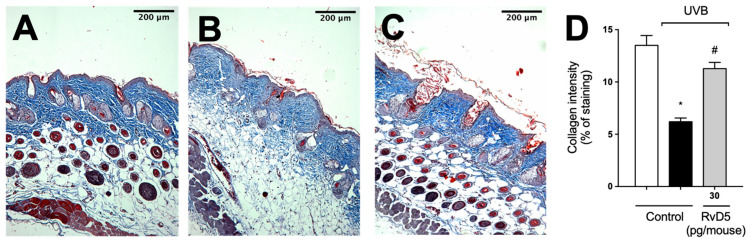
RvD5 inhibited UVB irradiation-induced collagen fiber degradation in the skin. Protocol was followed as depicted in Figure 1A to investigate collagen degradation using Masson’s trichrome staining. Representative images of the groups: (**A**) non-irradiated control treated with vehicle, (**B**) UVB irradiated treated with vehicle, and (**C**) UVB irradiated treated with 30 pg/mouse of RvD5 (100× magnification). Quantitative analysis of collagen degradation of experimental groups is presented as percentage of staining in panel (**D**). Bars are representative of two separate experiments and represent means ± SEM of 6 mice per group per experiment. Statistical analysis was performed by one-way ANOVA followed by Tukey’s test [* *p* < 0.05 compared to the non-irradiated control group; ^#^ *p* < 0.05 compared to the irradiated control group (vehicle)].

Given the deleterious effect of MMP-9 enzyme activity on collagen fibers, we quantitated collagen density in skin tissue sections stained with Masson’s trichome [73]. RvD5 reduced the degradation of skin collagen as observed by the preservation of the blue color in the Masson’s trichrome staining compared with the irradiated group (Figure 9). Therefore, the results of the enzymatic activity assay (MMP-9) and tissue staining (Masson’s trichrome) corroborate each other as well as the results on mast cells, neutrophils and macrophages (Figure 6 and Figure 7) [67,68,70].

### 3.9. RvD5 Inhibits UVB Irradiation-Induced Apoptosis of Keratinocytes

**Figure 10:**
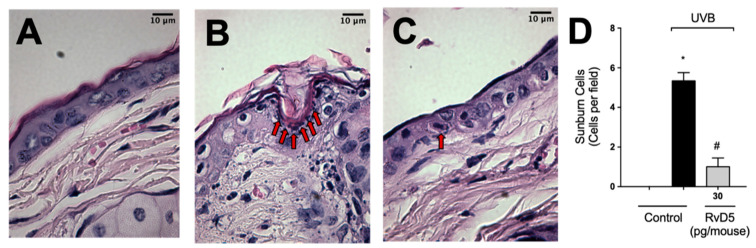
RvD5 reduces UVB radiation-induced sunburn cells. Mice were treated intraperitoneally with 30 pg of RvD5 1 h before and 7 h after the beginning of UVB irradiation. Sunburn cells were evaluated using hematoxylin and eosin staining (H & E) in skin samples collected 12 h after the end of irradiation. The sections stained with H & E were examined using light microscopy at 1000× magnification. Representative images of the groups: non-irradiated control (**A**), irradiated treated with vehicle (**B**), irradiated treated with 30 pg/mouse of RvD5 (**C**). Sunburn cells count is presented in cells per field in panel (**D**). Bars are representative of two separate experiments and represent means ± SEM of 6 mice per group per experiment. Statistical analysis was performed by one-way ANOVA followed by Tukey’s test [* *p* < 0.05 compared to the non-irradiated control group; ^#^ *p* < 0.05 compared to the irradiated control group (vehicle)]. Arrows indicate sunburn cells.

After high-level exposure to UVB radiation, keratinocytes undergo apoptosis (sunburn cells) [1] and cytokines such as TNFα are important in this phenomenon [13]. Sunburn cells were characterized histologically by chromatin condensation and eosinophilic cytoplasm [13]. UVB-irradiation increased sunburn cell count in comparison with the non-irradiated group (Figure 10). RvD5 treatment inhibited sunburn cell formation by 81% compared to the irradiated control group. These data indicate a protective effect of RvD5 upon UVB irradiation-induced keratinocytes apoptosis.

### 3.10. RvD5 Inhibits UVB Irradiation-Induced Epidermal Thickening

**Figure 11:**
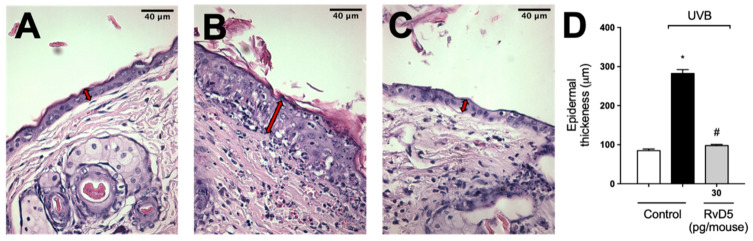
RvD5 reduced UVB irradiation-induced increase of epidermal thickness. Mice were treated intraperitoneally with 30 pg of RvD5 1 h before and 7 h after the beginning of UVB irradiation. The epidermal thickness was determined in samples collected 12 h after the end of irradiation and stained with hematoxylin and eosin staining (H&E). Representative images of the groups: non-irradiated control (**A**), irradiated treated with vehicle (**B**), irradiated treated with 30 pg/mouse of RvD5 (**C**). The epidermal thickness of experimental groups is presented in μm in panel (**D**). The sections stained with H & E were examined using light microscopy at 400× magnification. Bars are representative of two separate experiments and represent means ± SEM of 6 mice per group per experiment. Statistical analysis was performed by one-way ANOVA followed by Tukey’s test [* *p* < 0.05 compared to the non-irradiated control group; ^#^ *p* < 0.05 compared to the irradiated control group (vehicle)]. Arrows indicate the epidermal thickness.

Exposure to UVB radiation induces an increase in epidermal thickness due to the hyperproliferation of keratinocytes [74]. The histological analysis of hematoxylin and eosin-stained tissue sections indicated that epidermal thickness was significantly increased following exposure to UVB. On the other hand, epidermal hypertrophy was reduced by 93% compared to the irradiated control group when mice were treated with RvD5 30 pg/mouse (Figure 11).

## 4. Discussion

UVB irradiation triggers the overproduction of ROS causing oxidative stress [8] and inflammation [44]. RvD5 has been shown to enhance bacterial killing by increasing phagocytosis and ROS production [29]. An increase in ROS production would be detrimental in UVB irradiation skin inflammation, which is highly dependent on ROS [75], thus, whether RvD5 would have positive or negative effects in UVB skin pathology was unknown. Therefore, in the present study, we examined the effect of RvD5 on the inflammatory skin response following UVB irradiation. We observed that in UVB irradiation skin inflammation, which is a model of sterile inflammation, RvD5 reduced clinical signs of inflammation, such as skin edema, via mechanisms related to enhancing antioxidant endogenous responses. RvD5 also reduced the production of cytokines as well as the counts of inflammatory cells and pathological alterations in the skin. Therefore, our data reveal a hitherto unknown pharmacological activity of RvD5 in UVB irradiation skin sterile inflammation, which suggests its usefulness to prevent UVB-dependent pathological alterations in the skin.

In the experimental design, we considered that it would be important to test the activity of RvD5 in female mice because others have demonstrated RvD5 to be active in male, but not female rodents in the context of chemotherapy neuropathic pain and overt pain-like behavior triggered by formalin [38]. RvD5 could reduce disease parameters such as skin edema in female mice in the UVB inflammation skin model. Therefore, it was rational to continue the study using female mice since activity could be observed, and to limit the number of animals by selecting one gender only since our study was not aimed at searching for potential sexual dimorphism. Edema was first described as an inflammation clinical sign centuries ago, but remains a relevant disease parameter both in clinical and pre-clinical settings [12]. In this sense, we started our study by addressing whether RvD5 would present an effect against UVB-irradiation-triggered skin edema. RvD5 reduced skin edema. At the 12h time point, we can observe a reduction in endogenous antioxidants as assessed using the ABTS, FRAP and GSH methods [24,26,29]. These assays revealed that RvD5 prevented the loss of protective endogenous antioxidant of the skin with a reasonable pairing with skin edema and doses tested. Catalase activity is also an antioxidant enzyme since it converts hydrogen peroxide into water and molecular oxygen [76]. Again, RvD5 maintained the activity of this enzyme. Using the reverse approach, we observed that RvD5 reduced the production of superoxide anion, which is an initiating ROS in UVB irradiation [75] and lipid peroxidation, which is an end-product parameter of the oxidative stress in UVB irradiation [77]. Therefore, in terms of molecular events occurring in UVB irradiation pathology, RvD5 can reduce skin edema and oxidative stress, which is also consistent with the role of ROS in inducing edema [12].

The dose of RvD5 is low and was selected in a dose-response curve, 30 pg/mouse. It is unlikely that RvD5 would per se be an antioxidant molecule. Lipidic molecules such as vitamin E are antioxidants, but the dose ranges between 50 and 250 mg/kg [78,79]. In this sense, we speculated whether RvD5 would be capable of regulating endogenous antioxidant responses, which would explain the antioxidant activity observed upon RvD5 treatment. RvD5 enhanced the mRNA expression of Nrf2 and down-stream targets of this transcription factor such as NQO1 and HO-1 [60]. GSH production is also a result of Nrf2 activation [60] and we observed that RvD5, at least in part, prevented GSH depletion. Thus, the Nrf2 mRNA expression data is not a stand-alone result. It is in line with the NQO1 and HO-1 mRNA expression and GSH levels and other oxidative stress parameters that were quantitated. Furthermore, Nrf2 also leads to reduced production of ROS and cytokines by neutrophils and macrophages, resulting in inflammation reduction [61]. Therefore, in terms of mechanisms, RvD5 seems to actively regulate Nrf2-related antioxidant responses in vivo in UVB irradiation skin inflammation. This is somewhat unexpected since RvD5 enhances the neutrophil production of ROS and killing of *E. coli* [29]. However, there are differences between the disease mechanisms in each model. *E. coli* is a bacterium and causes an infection, thus, producing ROS is a necessary protection mechanism against this pathogenic agent [29]. RvD5 enhanced *E. coli* phagocytosis, and this might be a crucial cellular response that differentiates inflammation in infection and sterile inflammation. Phagocytosis activates the NADPH oxidase to produce superoxide anion inside the phagolysosome [80,81]. These properties of RvD5 in bacterial infection are important since they demonstrate that it is not immunosuppressive and rather enhances antibacterial responses [29]. In sterile inflammation, such as that triggered by UVB irradiation, there is no intrinsic role of phagocytosis to trigger superoxide anion production [80,81]. In UVB irradiation, in vitro, there is a fast production of superoxide anion within 3 min and peaking between 15–30 min [9]. This superoxide anion is produced by the complex I and III of the electron transport chain in mitochondria, which is a phagocytosis independent superoxide anion production mechanism [10,11]. In addition to the mitochondrial production of superoxide anion, cyclooxygenase and lipoxygenase produce this ROS as a by-product [12,13], and NADPH oxidase produces it as a primary product [7,8,15]. Thus, the main point is that superoxide anion production is an initiating mechanism of UVB irradiation skin inflammation, and occurs independently of the phagocytosis of pathogens, which might explain, at least in part, the differential effect of RvD5 in ROS production in *E. coli* infection versus UVB irradiation sterile inflammation [29].

In a similar manner to previous evidence in other models [32,38], RvD5 treatment reduced UVB-irradiation-triggered production of TNFα and IL-1β, which are pro-inflammatory cytokines. Again, a difference is that we used a model of sterile inflammation. It is also interesting that RvD5 reduced the production of IL-10 and TGFβ that act in UVB irradiation as anti-inflammatory cytokines. Our hypothesis is that as RvD5 reduced the inflammation, IL-10 and TGFβ production was not required to counterbalance pro-inflammatory cytokines [17]. Thus, in this acute sterile inflammation model, RvD5 did not act by enhancing anti-inflammatory cytokine production.

To better understand the phenotype changes caused by modulating oxidative stress and cytokine production, we needed to assess the profiles of cellular infiltration and pathological alterations in the skin upon UVB irradiation and RvD5 treatment. RvD5 reduced the counts of mast cells and LysM^+^ neutrophils/macrophages in the skin. These cells contribute to the production of pro-inflammatory ROS and cytokines [82]. RvD5 also changes the macrophage phenotype towards a non-inflammatory profile [83]. We did not assess this change in phenotype because such changes would occur at later time points in models with chronic repetitive exposition to UVB and not within 12 h. The present model is designed to study acute inflammatory and oxidative stress events in the skin [24,26,29,47,84]. To assess, for instance, changes in macrophage phenotypes towards anti-inflammatory-like profile, chronic exposure to UVB irradiation is more adequate [33] and is something to be pursued in the future based on the current results.

The combined effect of ROS and cytokines such as TNFα is responsible for inducing the enhanced activity of MMP-9 to degrade collagen as well as induce sunburn cells and thickening of the epidermal layer upon UVB irradiation [13,74,85]. Infiltrating cells such as neutrophils also help increase ROS and collagen degradation [12]. On the other hand, TGFβ has a role in tissue repair because it stimulates fibroblasts to produce collagen [64] and is secreted by anti-inflammatory-like macrophages [33]. Our interpretation is that as RvD5 induces endogenous antioxidant genes capable of bringing balance to the skin and avoiding the oxidative signaling and tissue damages upon UVB irradiation, RvD5 inhibited the characteristic UVB pathological events in the skin. By preventing the pathological events, the production of anti-inflammatory cytokines such as IL-10 and TGFβ, as well as the activity of TGFβ in tissue repair were not required. Nevertheless, it is possible that in chronic inflammatory conditions, RvD5 could be active depending on those anti-inflammatory conditions as long as there is sufficient time to switch macrophage phenotype to anti-inflammatory [83].

Comparing the activity of RvD5 with other SPMs in UVB irradiation of the skin, the first SPM to demonstrate activity against skin pathology caused by UVB irradiation was the lipoxin A4 (LXA4) [22]. This first study supported the investigation of the activity of BML-111, which is, as LXA4, an ALX/FPR2 receptor agonist [84]. It was also rational to investigate the activity of other SPMs such as RvD1 [23], aspiring-triggered RvD1 [25], and maresin 1 (MaR1) [24]. The overall activity of these SPMs is similar because they reduce inflammation and oxidative stress in the skin, limiting the skin pathology caused by UVB irradiation. However, the comparison among them is complex as well as defining which one would be better. This is because although some activities are shared (e.g., pro-resolution, anti-inflammatory, non-immunosuppressive and reduction of oxidative stress), which is rational since they belong to the same class of molecules, they have different receptors that in turn have variations in cellular expression. LXA4 and RvD1 are agonists of the ALX/FPR2 receptors while RvD1 is also an agonist of the GPR32 in humans. MaR1 is an agonist of the LGR6 and RvD5 of the GPR101 [86,87]. RvD5 has as an advantage that it reduces the requirement of antibiotics in bacterial infections, which is a point to be evaluated when there will be the need of chronic treatment or a condition in which the tissue can be infected [28,29,34]. RvD1 and MaR1 also present biological actions that help the host to deal with infections such as inducing bacterial phagocytosis and killing [28] while LXA4 impairs the control of *Staphylococcus aureus* infection in septic arthritis by dampening dendritic cell recruitment and function [88]. The definitive selection of the best SPM to treat UVB skin pathology may depend on additional factors such the development of pharmaceutical forms, further understanding biological roles based on their receptors as well as cost of production/dose needed. Considering the perspective of dose requirement, RvD5 was active at the picogram range while the other SPMs were active at the nanogram range in the current model of UVB irradiation of the skin. This lower dose of RvD5 than LXA4, RvD1, aspirin-triggered RvD1 and MaR1 [23,24,25] argues in favor of RvD5.

It is important to mention the limitations of the present study. We acknowledge that the Nrf2, NQO1 and HO-1 mRNA expression data would be improved by combining them with protein detection assays. Nevertheless, it is also suitable to highlight that NQO1, HO-1 and GSH are downstream targets of Nrf2, and the results on these molecules support the Nrf2 mRNA expression results as discussed above. GSH and cytokine production data also support the Nrf2 mRNA data [61]. Sunburn cell formation is a classic parameter in skin UVB irradiation; however, these results could also be improved by other methods assessing apoptosis. Another point is that the present study used only female mice. This choice was based on the fact that prior studies observed a lack of or reduced activity of RvD5 in female mice compared to male mice [38,39]. As far as we know, there is no previous study reporting RvD5 activity in females and lack of effect in males in the same experimental condition. This makes it reasonable to assume that if there would be reduced activity, it would be in females, not in males, guiding the choice to female mice. Lastly, the present study did not test the activity of RvD5 in naïve animals. One study performed such an approach. RvD5 did not alter nociceptive thresholds in naïve conditions, it was active solely when there was inflammatory or neuropathic pain [39]. In an in vitro study using human monocytic THP-1 cells, RvD5 did not alter cell viability up to 40 μM, which would be 2.88 micrograms per 200 μL for 1 × 10^5^ cells. There was no toxicity or alteration in cell viability and proliferation [27]. The selected dose of RvD5 in the present study was 30 pg per mouse. During peritoneal bacterial infection, RvD5 levels may vary from approximately 10 to 350 pg per peritoneal exudate [29], which reinforces that the dose applied in the present study is within the organism production capability. Although there is limited evidence on the activity of RvD5 in naïve conditions, these results indicate no major side effect. Furthermore, RvD5 reduces acute kidney injury induced by *Escherichia coli* lipopolysaccharide endotoxemia [40]. These data support that RvD5 would not induce organ lesion, but rather treat acute kidney injury.

In conclusion, to our knowledge, this is the first study to show that RvD5 reduces pathological changes in the skin caused by UVB irradiation. RvD5 mechanisms involved up-regulating Nrf2 expression and its downstream targets enhancing endogenous antioxidant protective responses and reducing inflammation. The present results support the rational development of RvD5 as a treatment to reduce UVB irradiation skin pathology opening a field that can involve compound modification, drug delivery systems and development of pharmaceutical formulations bearing RvD5 as an active molecule.

## Data Availability

The data that support the findings of this study are available from the corresponding author upon reasonable request.
